# Automated mass action model space generation and analysis methods for two-reactant combinatorially complex equilibriums: An analysis of ATP-induced ribonucleotide reductase R1 hexamerization data

**DOI:** 10.1186/1745-6150-4-50

**Published:** 2009-12-09

**Authors:** Tomas Radivoyevitch

**Affiliations:** 1 Department of Epidemiology and Biostatistics, Case Western Reserve University, Cleveland, Ohio 44106, USA

## Abstract

**Background:**

Ribonucleotide reductase is the main control point of dNTP production. It has two subunits, R1, and R2 or p53R2. R1 has 5 possible catalytic site states (empty or filled with 1 of 4 NDPs), 5 possible *s*-site states (empty or filled with ATP, dATP, dTTP or dGTP), 3 possible *a*-site states (empty or filled with ATP or dATP), perhaps two possible *h*-site states (empty or filled with ATP), and all of this is folded into an R1 monomer-dimer-tetramer-hexamer equilibrium where R1 j-mers can be bound by variable numbers of R2 or p53R2 dimers. Trillions of RNR complexes are possible as a result. The problem is to determine which are needed in models to explain available data. This problem is intractable for 10 reactants, but it can be solved for 2 and is here for R1 and ATP.

**Results:**

Thousands of ATP-induced R1 hexamerization models with up to three (*s*, *a *and *h*) ATP binding sites per R1 subunit were automatically generated via hypotheses that complete dissociation constants are infinite and/or that binary dissociation constants are equal. To limit the model space size, it was assumed that *s*-sites are always filled in oligomers and never filled in monomers, and to interpret model terms it was assumed that *a*-sites fill before *h*-sites. The models were fitted to published dynamic light scattering data. As the lowest Akaike Information Criterion (AIC) of the 3-parameter models was greater than the lowest of the 2-parameter models, only models with up to 3 parameters were fitted. Models with sums of squared errors less than twice the minimum were then partitioned into two groups: those that contained no occupied *h*-site terms (508 models) and those that contained at least one (1580 models). Normalized AIC densities of these two groups of models differed significantly in favor of models that did not include an *h*-site term (Kolmogorov-Smirnov p < 1 × 10^-15^); consistent with this, 28 of the top 30 models (ranked by AICs) did not include an *h*-site term and 28/30 > 508/2088 with p < 2 × 10^-15^. Finally, 99 of the 2088 models did not have any terms with ATP/R1 ratios >1.5, but of the top 30, there were 14 such models (14/30 > 99/2088 with p < 3 × 10^-16^), i.e. the existence of R1 hexamers with >3 *a*-sites occupied by ATP is also not supported by this dataset.

**Conclusion:**

The analysis presented suggests that three *a*-sites may not be occupied by ATP in R1 hexamers under the conditions of the data analyzed. If *a*-sites fill before *h*-sites, this implies that the dataset analyzed can be explained without the existence of an *h*-site.

**Reviewers:**

This article was reviewed by Ossama Kashlan *(nominated by Philip Hahnfeldt)*, Bin Hu *(nominated by William Hlavacek) *and Rainer Sachs.

## Background

### Introduction

**Figure 1:**
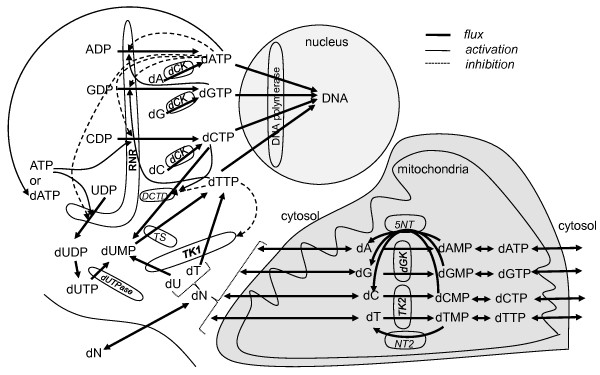
**The dNTP supply system**. Thick lines are fluxes, thin solid lines are activations, thin dashed lines are inhibitions. Key enzymes are described in the text. An RNR *s*-site mediated large positive feedback loop ATP → dCTP → dUMP → dTTP → dGTP → dATP terminates when dATP binds the R1 *a*-site to inhibit all 4 RNR reductions. Models of enzymes of this system will eventually be useful in anticancer drug dose time course optimizations [1].

The dNTP supply system produces dNTPs at rates that match those demanded by DNA replication and repair. With respect to ribose ring moieties, it is comprised of both a *de novo *system whose substrates are ribonucleoside diphosphates (NDPs) and a salvage system whose substrates are deoxynucleosides (dNs). The *de novo *system includes ribonucleotide reductase (RNR), deoxycytidylate deaminase (DCTD), and thymidylate synthetase (TS), and the salvage system includes deoxycytidine kinase (dCK), thymidine kinase 1 (TK1), deoxyguanosine kinase (dGK) and thymidine kinase 2 (TK2), see Fig. 1. The dNTP supply system is important because many anticancer agents target or traverse it (e.g. gemcitabine, hydroxyurea, triapine, 5-FU) or damage DNA directly (e.g. ionizing radiation, alkylating agents, oxaliplatin) and thus place demands on it for replacement dNTPs. In the future, mathematical models of cancer relevant systems will be needed to optimize multi-agent anticancer dose timings [1]. For example, gemcitabine (dFdC, diflourodeoxycytdine) [2] absorption is rate limited by dCK [3,4], dFdC targets RNR [5], dFdC resistance is associated with RNR over expression [6,7], and differential ionizing radiation (IR) sensitivity that dFdC imparts onto mismatch repair (MMR) defective cells may be due to mismatches caused by dNTP pool imbalances caused by RNR inhibition, rather than differential dFdC incorporation into DNA [8], so mathematical models of dNTP supply will be needed to optimize dFdC-IR therapies of MMR defective cancers; MMR defective cancers are significant as they comprise ~10% of colorectal [9], gastric [10], pancreatic [11], urinary [12], gynecologic [13,14] and glioma [15] cancers.

The dNTP supply system is ideal for cancer systems biology research because, among cancer relevant processes, it is perhaps the best understood. This is important because, intuitively, the more understanding a mathematical model captures, the more likely it is to be more useful than a conceptual model. Thus, the dNTP supply system is well poised to be successfully controlled better with mathematical modeling than without, and because of this, this system could become a standard of success in systems biology; the basis of this argument is prior success in the use of mathematical models to improve the control of well understood systems such as power plants and airplanes.

RNR (NDP → dNDP) [16] has two subunits, R1, and R2 or p53R2 [17,18]. On short time scales of seconds to minutes, RNR is controlled through two R1 regulatory sites, a selectivity (*s*-) site that is somewhat analogous to a radio tuning control knob, and an activity (*a*-) site that can be thought of as a volume control knob. Complicated positive and negative dNTP-mediated feedback loops (Fig. 1) impinge upon these two sites to implement a sophisticated solution to a challenging dNTP pool balance regulation problem; if pool balance regulation performance varies across individuals and MMR performance also varies, individuals compromised in both systems may be predisposed to cancer [19]. RNR functional complexity is mirrored by the combinatorial complexity of its R1 subunit: its catalytic site can be empty or filled with 1 of 4 NDP substrates, its *s*-site can be empty or filled with ATP, dATP, dTTP or dGTP, its *a*-site can be empty or bound by ATP for activation or dATP for inactivation, it may have an *h*-site that can be empty or bound by ATP [20], and all of this is folded by an R1 monomer-dimer-tetramer-hexamer equilibrium where R1 j-mers may also be bound by variable numbers of R2 (or p53R2) dimers. As a result, trillions of R1 complexes are possible if R1, R2, UDP, CDP, GDP, ADP, ATP, dATP, dTTP and dGTP are all present (in this case ~10^2 ^R1 monomers implies ~10^12 ^R1 hexamers) and the problem then is to determine which are needed in models to explain the data at hand. To appreciate the magnitude of the problem, if 10^12 ^complexes are possible, the number of possible complete dissociation constant models is 2 raised to the 10^12 ^(i.e. 1 followed by ~300 billion zeros), since each complex, and its corresponding complete dissociation constant K, can either be in the model (estimated) or out (set to infinity if the model hypothesizes that the concentration of the complex is approximately zero across all of the experimental conditions of the dataset). This huge number of models is even greater if, in addition to hypotheses that complete dissociation constants are infinite, hypotheses that binary dissociation constants equal each other are also considered. Though this problem is intractable for 10-reactants, 2-reactant solutions are feasible and may yield insights needed to enable 3-reactant solutions, and so on.

The R [21] package Combinatorially Complex Equilibrium Model Selection (ccems) [22] is used here to automatically generate thousands of ATP induced R1 hexamerization models partitioned into two classes: those that include model terms of complexes with ATP occupied *h*-sites (i.e. models that would support the existence of *h*-sites if selected) and those that do not. Comparisons of the Akaike Information Criterion (AIC) [23,24] of these two classes of models were then used to assess the extent of *h*-site evidence strictly in the ATP-induced R1 hexamerization dynamic light scattering (DLS) data in figure 1 of reference [20]. No evidence was found. As discussed in Kashlan's review below, evidence for an *h*-site may, however, exist under different experimental conditions.

### A Simple Example

To introduce concepts of model space generation, standard models of competitive and non-competitive inhibition are derived below as instances of models in two systematically defined model spaces, one of spur graphs which focus on *complete *dissociation constants and hypotheses that they are infinite, and one of grid graphs which focus on *binary *dissociation constants and hypotheses that they are equal.

#### Spur Graphs

**Figure 2:**
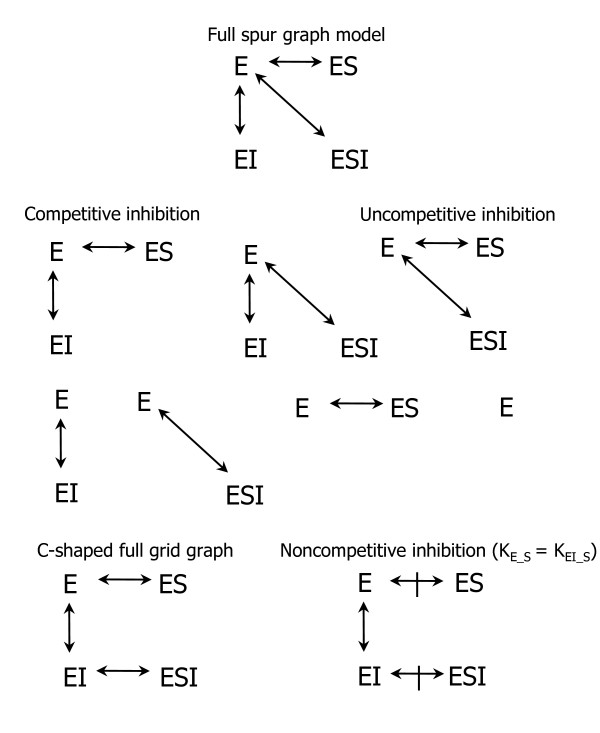
**ESI model graphs**. The full spur graph at the top generates the seven models/graphs below it via hypotheses taken one at a time, two at a time, etc, that dissociation constants are infinite. The C-shaped grid graph is a data-fitting equivalent of the full spur graph. It generates the non-competitive inhibition model to its right where parallel edge binary K's are equal.

Consider the enzyme-substrate-inhibitor (ESI) models of Fig. 2. The full spur graph at the top of this figure is represented by the following total concentration constraint (TCC) system of coupled free concentration polynomials:(1)

where [E_T_], [S_T_] and [I_T_] are the known total concentrations, or system inputs, [E], [S] and [I] are model dependent free concentrations, which can be thought of as predicted latent system state variables, the dissociation constants *K *are *complete *dissociation constants, and implicit in these equations is the equilibrium concept of mass action, e.g. [ESI] = [E][S][I]/K_ESI_; in spur graphs every complex is reached from free E in a single step and thus every term in the corresponding equations has only one *K *parameter in its denominator, see [25]. The model in Eq. (1) is called a full model because it is fully parameterized to the extent that no constraints have yet been placed on its parameters. Model spaces are then generated from full models by applying constraints to them. For example, the other spur graphs in Fig. 2 are obtained from the full spur graph by applying *K *= ∞ constraints to remove nodes in the graph, and thus columns of *K *terms in Eq. (1), one at a time, two at a time, and three at a time. Each resulting graph/model is then a hypothesis that some *K*'s are approximately infinitely large, or that concentrations of the deleted complexes are so small, throughout all of the experimental conditions of the dataset, that they can be approximated as zero, e.g. the competitive inhibition model, where substrate and inhibitor bind the same site and thus cannot bind simultaneously, hypothesizes that [ESI] = 0, or equivalently, that *K*_ESI _= ∞, and its equations are

Note that this model corresponds to the full spur graph less the ESI node/edge and that it can also be viewed as the pair (*K*_ESI _= ∞, Eq. 1).

#### Grid Graphs

Figure 2 also shows a C-shaped full grid graph with system equations(2)

where underscores in subscripts indicate specific binary reactions. In grid graphs, because the dissociation constants are binary, equation terms that represent complexes of *n *reactants have *n *- 1 *K *parameters in their denominators.

There is a one-to-one mapping between the *K*'s in grid graph system (2) and those in spur graph system (1), namely, *K*_ES _= *K*_E_S_, *K*_EI _= *K*_E_I_, and *K*_ESI _= *K*_E_I_*K*_EI_S _or *K*_EI_S _= *K*_ESI_/*K*_EI_. It follows then that (1) and (2) are data-fitting equivalents. The added value of full grid graph systems such as system (2) is their ability to spawn new hypotheses that cannot be generated by corresponding full spur graph systems such as (1). The additional hypotheses are binary *K *equality hypotheses. In the example here there is only one such hypothesis/model, the non-competitive inhibition model *K*_E_S _= *K*_EI_S _where inhibitor binding has no detectable effect on substrate binding.

#### Solutions

System equations are solved as the steady state of a parent system of ordinary differential equations (ODEs) [25], e.g. system (1) is solved by simulation of(3)

to large τ where τ has nothing to do with real time and the state trajectory is merely algorithmic and thus not a biophysical path. Free concentration solutions then map to complex concentrations via mass action laws and these are then mapped to expected measurements, e.g. see Eq. (6) below and Eqs. (16-18) in [25].

## Results

### Limitations

The methods presented here are currently limited to biochemical systems where one central hub protein mediates all of the interactions and total concentrations of the reactants are approximately known exactly. It is assumed that the latter condition is adequately met in analyses of data derived from systems that were reconstituted from purified reactants. If the hub protein has more than one binding site for the same ligand, as R1 does for ATP, to interpret model terms, a specified sequence of site filling must be assumed. This assumption, made due to lack of a better option, may not hold. Automated model space generation is currently limited to two-reactant systems.

### ATP-induced R1 Hexamerization Models

#### Full model

To generate a space of ATP induced R1 hexamerization models, the first step is to pick a full model and the second step is to apply *K *hypotheses to it [25]. Full models that include *s*, *a *and *h *ATP binding sites generate two classes of models: those that include at least one occupied *h*-site complex and that thus support the existence of an *h*-site, if selected, and those that do not, i.e. those that allege that all occupied *h*-site complex concentrations are approximately zero and that thus support claims that the *h*-site is not needed to explain the data, if selected. The full model below generates both of these model types.

Full models place an upper bound on the complexity and size of the model space and should thus be no more complicated than needed to answer the question of interest, e.g. models with a fourth ATP binding site should not be considered as there is no evidence that such a site exists. Lower bounds can also be placed on the simplicity of complexes, and this too can reduce the size of the model space. Thus, based on the crystal structure of yeast R1 dimers that shows that the *s*-site is created at the R1 dimer interface [26], and based on dTTP induced R1 dimerization being well represented by free reactants forming (R1)_2_(dTTP)_2 _directly [25] (dTTP binds only at the *s*-site), it is reasonable to assume that R1 oligomer *s*-sites are always fully occupied (i.e. that oligomers cannot form without full *s*-site occupancy) and that R1 monomer *s *sites are always unoccupied (i.e. that the *s*-site does not exist in R1 monomers). With these restrictions, denoting ATP and R1 by X and R respectively, and using(4)

the full spur graph system equations are:(5)

where, in each equation, first sum limits assume *s*-sites cannot be bound in monomers and other sum limits assume *s*-sites must be bound in oligomers; here X = ATP is used because a = dATP and A = ADP are being reserved for subsequent RNR models and R^j^X^i ^is used instead of R_j_X_i _to stress connections to polynomials.

#### Interpretations

The hub protein monomer complex RX can be interpreted as X bound to either the *a*- or *h*-site. Because the *a*-site is known to exist, *a*-site binding will be assumed. RXX is then a monomer with both the *a*- and *h*-sites occupied. For oligomers, in addition to all of the *s*-sites being prefilled, it is assumed that: R^2^X^2 ^through R^2^X^4^, R^4^X^4 ^through R^4^X^8^, and R^6^X^6 ^through R^6^X^12^, have zero to full *a*-site occupancies and no *h*-site occupancies, and that R^2^X^5 ^and R^2^X^6^, R^4^X^9 ^through R^4^X^12^, and R^6^X^13 ^through R^6^X^18^, have partial to full *h*-site occupancies in addition to completely filled *a*-sites (and *s*-sites). Model inferences will be based on these interpretations.

#### Output linkage

The fitted output measurements are mass-weighted average protein masses(6)

where [*R*_jT_] is the total *j*-mer concentration (i.e. the sum of the concentrations of all *j*-mer hub protein complexes),

is the total hub protein concentration, M_1 _is the mass of a monomer (90 kDa for the R1 subunit of RNR), *ε *is noise with constant variance and zero mean, and the *j*^2 ^in the numerator includes one factor of *j *because the mass of a *j*-mer is *j *times that of a monomer, and another factor of *j *because light scattering is proportional to mass; ligand masses are treated as negligible relative to protein masses.

#### Implementation

Nonlinear least squares was used to fit the models. The fitted models were then rank ordered by their AICs [24]. Because the number of data points *N *is small at 15, the small sample size corrected version of the AIC was used: AIC = 2*P + 2*P(*P*+1)/(*N*-*P*-1) + N*log (2π) + N*log (SSE/*N*) + *N *where *P *is the number of estimated parameters (including the variance) and SSE is the sum of squared errors [24]. In parameter optimizations (i.e. SSE minimizations, see Methods) the initial complete dissociation constant values were 100 μM raised to the sum of the powers of the numerators in Eq. (4) minus one, i.e. *j *+ *i *- 1. This was critical, as it increased the number of models that converged from roughly 10% (when 1 μM was used uniformly) to nearly 100%. Models were fitted in parallel in a load balanced manner using R [21]; the R package ccems uses the R package snow (small network of workstations) to accomplish this. R scripts that were used to produce the results in this and the accompanying paper are available as examples in the papers directory of ccems.

#### Spur graphs

The number of complexes represented in Eq. (5) is 2 + 5 + 9 + 13 = 29 and this implies that the number of spur models is 2^29 ^= ~500 million. Relevant here, however, is the number of 1-, 2- and 3-parameter models. There are 29 single-edge models, 406 (29 choose 2) two-edge models, and 3654 (29 choose 3) three-edge models. The lowest AIC of the 3-parameter models (144.4) was higher than the lowest AIC of the 2-parameter models (142.7), so 4-parameter models were not fitted; as parameter numbers increase AICs typically first decrease as SSEs decrease, but then rise and continue to rise due to over-parameterization.

#### Grid graphs

Binary dissociation constants that are alleged equal to one another must be defined on a per site basis in terms of *k*_off_/*k*_on_. For example, using the equilibrium property that net fluxes between any two complexes must vanish, for *a*-sites in tetramers, the on and off fluxes of the 1^st ^ligand yield

where factors of 1 and 4 arise because there is 1 occupied site for the dissociation reaction and 4 unoccupied sites for the association reaction, respectively. Similarly, for second, third and fourth ligand bindings to a tetramer *a*-site, the per site binary dissociation constants *K *are:

These binary *K *can be hypothesized to equal each other and same-site per-site binary *K *of other oligomers such as

Similar arguments apply to *h*-sites, with *X*^*i*+*j *^replacing *X*^*i *^in *j*-mers.

**Figure 3:**
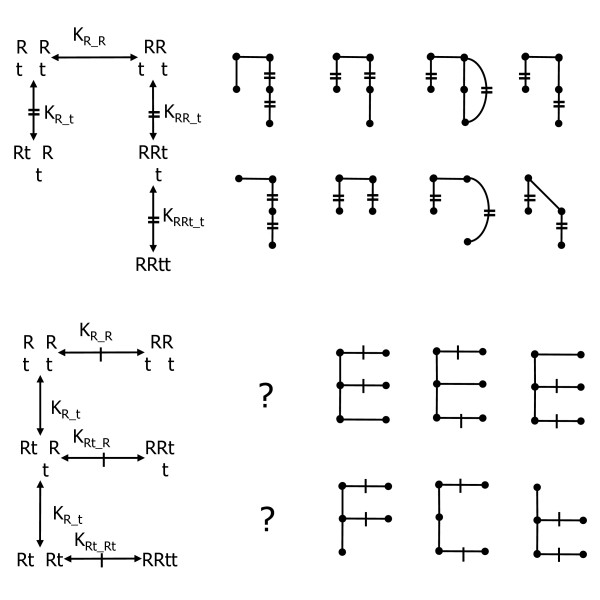
**dTTP-induced R1 dimerization K equality models**. The n-shaped graphs are equal to their corresponding E-shaped graphs below them. The rightmost three columns are very unlikely (see text). R = R1, t = dTTP and edges marked = or -- are alleged equal.

**Figure 4:**
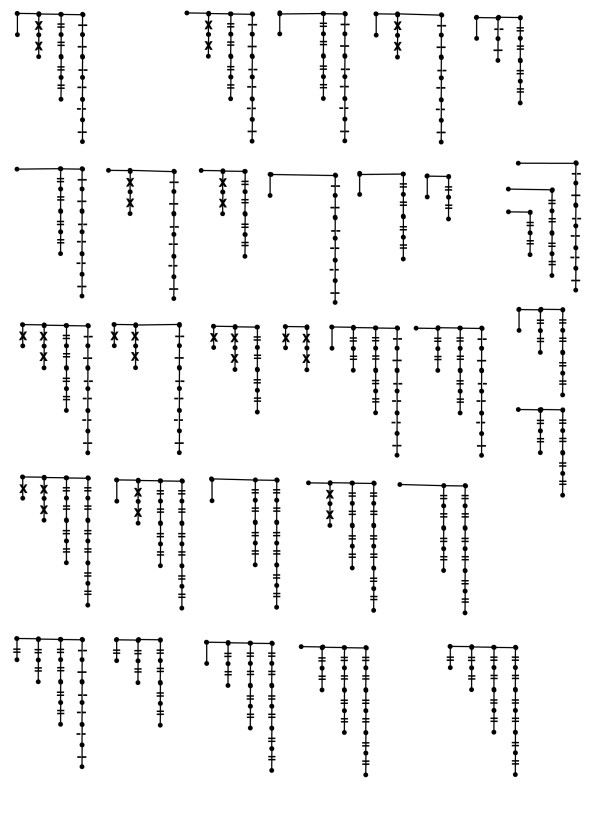
***K *equality RX model space**. The graphs shown are the same for both *a*- and *h*-site models. The top two rows have independent threads and the bottom two rows have at least two threads that have equal binary *K *values indicated by =, --, or x. Bridge edges in the horizontal bars of each graph (i.e. the curtain rods) are spur edges from the hub, rather than binary K. Left to right, threads on curtain rods correspond to monomers, dimers, tetramers and hexamers.

To introduce a few additional concepts in their simplest forms, the next two paragraphs consider dTTP induced R1 dimerization. That not all *K *equality hypotheses take the form *K *= *K*' is seen in the penultimate column of the n-shaped graph pairs in the top half of Fig. 3 where K^2^_R_t _= [R]^2^[t]^2^/[Rt]^2 ^= K_RR_t_K_RRt_t _= [RR][t]^2^/[RRtt]; here t denotes dTTP. This graph pair can, however, be restated as the equivalent *K *= *K*' E-shaped graph pair shown in the corresponding positions below it. Though earlier work focused on E-shaped graphs [25], based on the paragraph that follows, it suffices to consider only n-shaped graphs and natural extensions thereof (e.g. see Fig. 4).

The first two columns of *K *equality models/hypotheses in Fig. 3 are plausible, as a protein could be so rigid that a ligand binding site is unchanged with respect to binding affinity regardless of other bindings (first column) and this could be true within *j*-mers but not between them (second column). The third column hypotheses are less likely, however, as they claim that binding of R (to R), which is massive relative to ligand and thus more likely to cause alterations upon binding, causes no change in ligand site affinities, yet, binding of the first small ligand to the dimer alters the dimer structure enough to change ligand binding at the second site. Continuing, fourth column hypotheses are even less likely, as they claim that the first ligand binds dimer differently than monomer, yet, after it binds, by chance, the second ligand binding energy exactly equals the amount needed for the product of these *K*'s to equal the square of the monomer ligand *K*. Equivalent E-shaped graphs (same column) support this claim of unlikelihood, as they claim that although dimerization energies are different between R + R and Rt + R, they somehow return to the R + R value when both reactants are Rt. Finally, in the fifth column, it would be remarkable if ligand binding to free dimer differs from ligand binding to free monomer, yet somehow, binding of the first ligand returns the unoccupied dimer subunit to a state indistinguishable from that of the free monomer (with respect to its ligand binding affinity). The third and fifth columns can be interpreted in terms of rigid asymmetric dimers where one subunit holds its monomer shape and the other has a different shape with either tighter (fifth column) or weaker (third column) ligand binding. From this perspective, it is very unlikely that all of the dimerization induced shape changes (deformation energies) would fall strictly onto one of two identical subunits. Thus, the grid model space used here will only include *K *equality hypotheses that are analogous to the first 2 columns in Fig. 3, and it suffices to consider n-shaped graphs.

Within a site type, binary *K*'s of *j*-mers can be depicted as threads hanging from a curtain rod as shown in Fig. 4. In the accompanying paper, binary *K *equalities in contiguous stretches within threads are considered. Here, each thread is homogeneous in its binary *K *value (i.e. only full thread length contiguous stretches are considered) and *K *equality models are instead generated by considering thread *K *values as independent of other threads (top 2 rows in Fig. 4), infinite (graphs with missing threads), or equal to those of other threads of the same site type (bottom 3 rows in Fig. 4) within contiguous stretches of threads, the idea being that if one protein oligomerization step alters a ligand *K*, it is unlikely that an additional step would return it to one of its previous values.

Since R binds R to form R^2 ^via one protein surface, and since it is likely that R^2 ^binds R^2 ^and R^4 ^using two different protein surfaces (or patches thereof), no hypotheses of *K *equivalence will be considered between the saturated *s*-site complexes R^2^X^2^, R^4^X^4 ^and R^6^X^6 ^(i.e. complexes in the curtain rods in Fig. 4). Thus, all of the *K *equality hypotheses explored will be with respect to ligand binding site constants in threads.

The binary *K *equality model space of interest here is the product of a space of *a*-site models and a completely analogous space of *h*-site models. The 32 models shown in Fig. 4 thus imply a *K *equality space of 1024 models. If thread head nodes within curtain rods are allowed to remain in models where all other nodes in the same thread have infinite *K*, the number of models increases: models missing hexamer threads split into two models (there are 8 of these in Fig. 4) and models missing both tetramer and hexamer threads (there are 3 of these in Fig. 4) split into four models. The total number of grid models is then (32 + 8 + 9)^2 ^= 49^2 ^= 2401.

#### Models that contain hexamer terms

Since external data [27] confirms ATP induced R1 hexamerization, the model space was reduced to only models that contain at least one hexamer term. This reduced the number of grid models with 2 and 3 parameters to 2 and 15 (from 7 and 36) and the number of spur models with 1, 2 or 3 parameters to 13, 286 and 3094 (from 29, 406 and 3654), i.e. the number of models is now 17 + 3393 = 3410.

#### Competitive models

**Figure 5:**
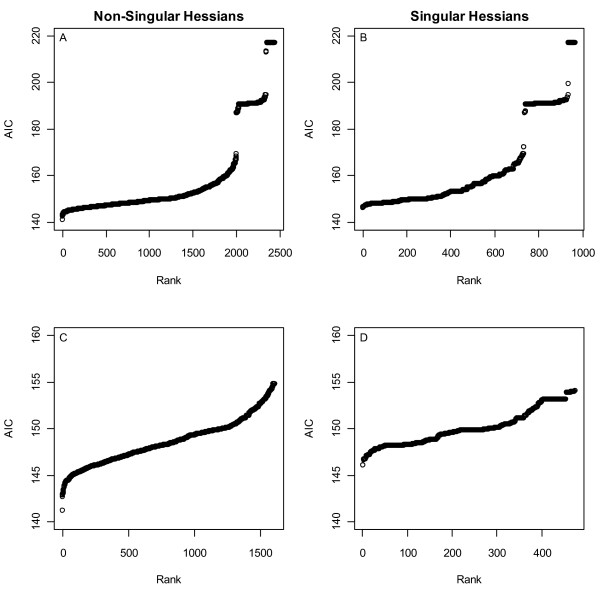
**AIC model densities versus AIC model rank**. **A) **and **C) **show models that had non-singular Hessians (matrices of objective function second derivatives) at their optimums; **B) **and **D) **are models with singular (determinant = 0) Hessians, i.e. models that converged onto likelihood surfaces that were flat in one direction. **C) **and **D) **show 1613 and 475 models with SSEs < twice the minimum SSE.

Of 3410 ATP-induced R1 hexamerization models automatically fitted to the DLS data [20], four failed to converge (these all involved X^17 ^and X^18^), 966 (Fig. 5B) converged but had singular Hessians (12 and 954 of these were 2- and 3-parameter models and all had infinite upper confidence limits in all parameters) and of the remaining 2440 fits (Fig. 5A), 1200 had no infinite upper bounds, 680 had 1 of 3, 91 had 1 of 2, and 369 had 2 of 3. Models with no infinite upper bounds comprised 64%, 6% and <1% of the lowest to highest AIC clusters shown in Fig. 5A. To purge the space of problematic models (e.g. those that were either incorrect or sensitive to initial parameter values), the space was reduced to models with SSEs that were less than twice the minimum SSE of the fitted models. AICs of the resulting 2088 models are shown in Figs. 5C and 5D.

#### h-site existence

**Figure 6:**
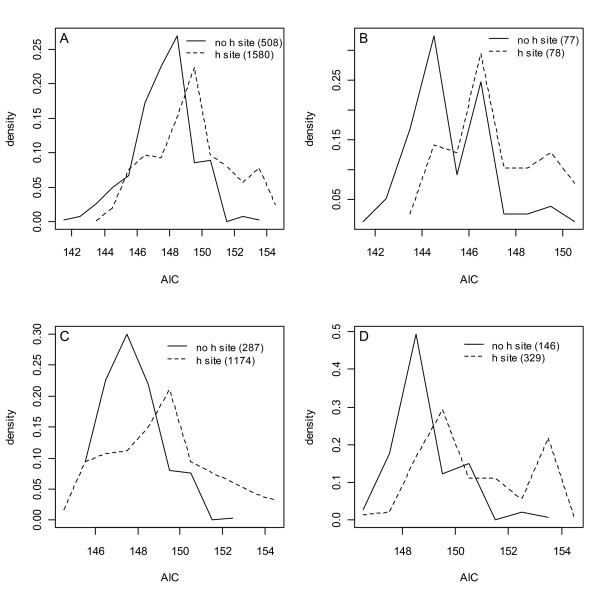
**Normalized AIC densities of models with SSEs less than twice the minimum**. **A) **The models of Figs. 5C and 5D. There are 508 (1580) models without (with) an occupied *h*-site. **B) **The 1- and 2-parameter models of A). **C) **The 3-parameter models of A). **D) **The models in A) that have singular Hessians (i.e. the 475 models in Fig. 5D). In all cases a difference in *h*-site hypothesis densities is supported by a two-sample Kolmogorov-Smirnov test, P < 10^-15 ^(A, C, D) and P < 10^-5 ^(B).

Fig. 6A shows normalized AIC densities of the models in Figs. 5C and 5D partitioned into two groups, those that do not represent any complexes that have occupied *h*-sites (508 models) and those that do (1580 models). That these densities differ is apparent by inspection, a Kolmogorov-Smirnov p < 10^-16 ^and by 28 of the top 30 models not including an *h*-site term, i.e. 28/30 > 508/2088 with p < 1 × 10^-15^. The densities were then decomposed into models with <3 parameters (6B), 3 parameters (6C) and singular Hessians (6D). In each case the same conclusion held, the DLS data did not support the existence of an *h*-site.

**Figure 7:**
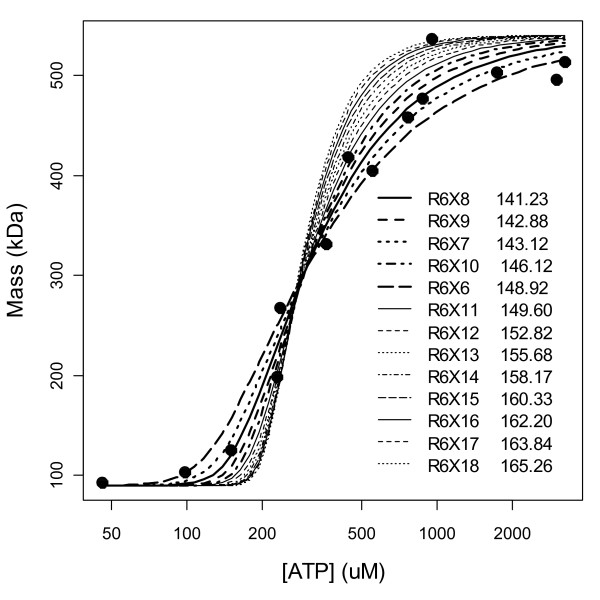
**Fits of the 1-parameter models**. The legend in the plot indicates the model order ranked by AICs (values shown). The top 5 models are indicated by thicker lines. Beyond 5 or more *a*-sites occupied by ATP and with increasing numbers of *h*-sites occupied, the fits become worse and worse.

Fig. 7 shows the fits of only the 1-parameter models. The model R^6^X^8 ^fits the data the best and is immediately flanked by R^6^X^7 ^(less steep) and R^6^X^9 ^(steeper) which also fit reasonably well, but beyond these, the fits become noticeably poorer; since these models all have the same number of parameters, AICs in the legend reflect SSEs and the gap between the 3^rd ^and 4^th ^model thus reflects a lack of fit. That R^6^X^6 ^is a poor fit supports the assumption that *s*-sites are pre-filled, and that R^6^X^10 ^is a poor fit supports a hypothesis that only 1 to 3 hexamer *a*-sites are filled. That R^6^X^11 ^and higher models provide worse and worse fits with increasing numbers of bound ATP supports the Fig. 6 conclusion that this dataset does not support the existence of an *h*-site.

**Figure 8:**
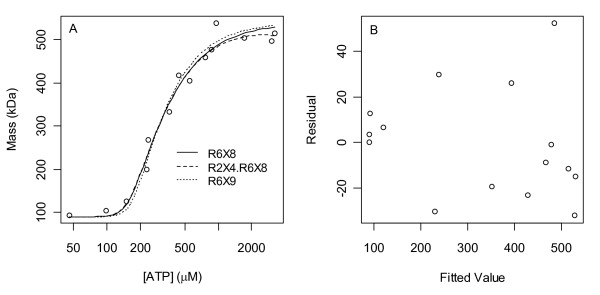
**Top 3 fits to the data of Kashlan *et al***. [20]. **A) **Fits of the top 3 models. The second model uses its dimer term to capture a slight downturn in average mass at high [ATP] (see Fig. 9), consistent with its geometric mean binding constant being greater than that of the hexamer term in Table 1. Not shown in this plot is the point (0 μM, 90 kDa) which all models fit perfectly if M_1 _= 90 is fixed (as it is here) rather than estimated. **B) **Residuals of the top model R6X8. The reduced variance and positive mean of the first 4 residuals may be due to bias arising from prior knowledge that the monomer is 90 kDa and thus too prior knowledge that the average mass must increase from 90 kDa. Non-weighted least squares gives less weight to these 4 points which, coincidentally, is advantageous given these suspicions. PlotDigitizer (Methods) was used to obtain the data from a pdf of the original paper, and as this step involves human intervention, it too may have introduced some bias and random error.

**Figure 9:**
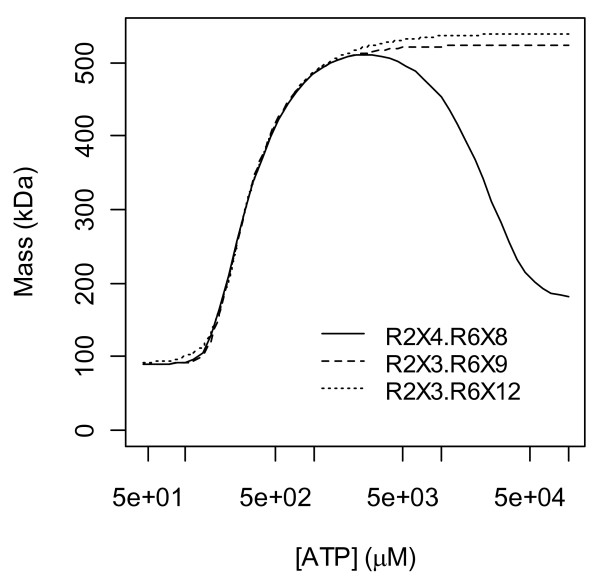
**Model limits at large ligand concentrations**. In the second model in Fig. 8 and Table 1 (solid line here) the dimer term R2X4 causes a below expectation peak (510 instead of 540) at high [ATP]. In the limit of very high [ATP], this model is dominated by the R2X4 term (average mass approaches 180 kDa) because this term partitions more X into a bound state with R than the hexamer R6X8, i.e. 4/2 = 2 > 8/6 = 1.3. These ratios are both 1.5 in the model R2X3.R6X9 (dashed line) which has a balanced population in this limit (with a limiting average mass of 523 kDa). These ratios are 1.5 and 2 in the model R2X3.R6X12 (dotted) which yields pure hexamers (average mass = 540 kDa) in the limit of infinite ATP.

**Figure 10:**
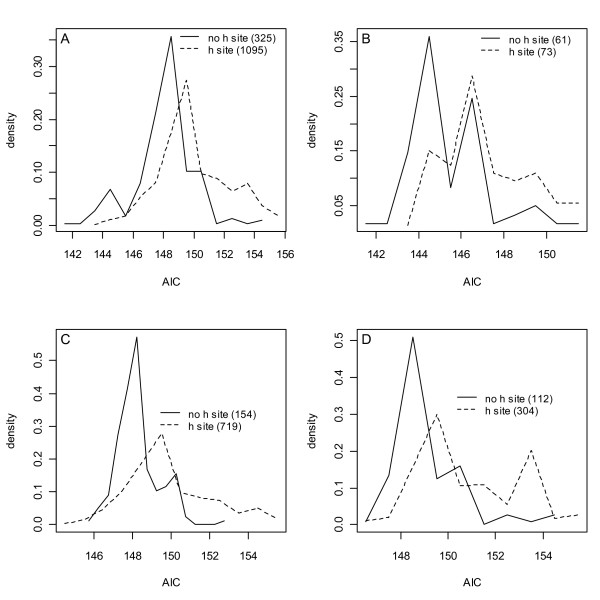
**Normalized model number densities of AICs of models with SSEs less than twice the minimum SSE and monotonic non-decreasing ratios of ATP per R1 as oligomer sizes increase**. **A) **The complete set of such models. **B) **The 1- and 2-parameter models. **C) **The 3-parameter models. **D) **Models with singular Hessians. In all cases a difference in *h*-site hypothesis densities is supported by a two-sample Kolmogorov-Smirnov test, P < 10^-15 ^(A, C, D) and P < 2 × 10^-5 ^(B). Compare to Fig. 6.

Fits of the top 3 models are shown in Fig. 8 and their parameter estimates are given in Table 1. The second of these is a 2-parameter model that differs from the other two in that it uses its second parameter to avoid its obligation to reach an average mass of 540 kDa in the limit of large [ATP]; when this model was extrapolated to [ATP] = 1 M (Fig. 9) the predicted average mass in this limit was 180 kDa. It was conjectured then that complexes with the highest ratios of ATP bound per R1 completely dominate the distribution in the limit of high ligand concentrations and that only in cases of maximum ratio ties does a balance result (i.e. that the system's objective in this limit is to partition as much ATP as possible away from its free form and into a bound form). Representative models of both a balance and of hexamer dominance support this conjecture (Fig. 9). If it is asserted then that the ATP per R1 ratio cannot decrease as higher R1 oligomers are formed, the space of 2088 models reduces to 1420 models, but the calculations of Fig. 7 still yield the same conclusion, i.e. no *h*-site existence (Fig. 10). If the ratio is forced to strictly increase with oligomerization, the number of models is 1287, and again, the same conclusion holds (plots not shown).

**Table 1:** The top 3 models (lowest AIC) of the RX model space.

Model	Parameter	Initial Value	Optimal Value	Confidence Interval
**1 IIIIIIIIIIIJIIIIIIIIIIIIIIIII**	R6X8	100.000^13	63.101^13	(59.878^13, 66.175^13)

	SSE	840487.830	7726.693	

	AIC	211.573	141.234	

	CPU	0.000	3.465	fit succeeded

**2 IIIIIJIIIIIJIIIIIIIIIIIIIIIII**	R2X4	100.000^5	432.997^5	(311.064^5, 601.845^5)

	R6X8	100.000^13	62.796^13	(59.878^13, 66.175^13)

	SSE	778605.476	6873.411	

	AIC	213.608	142.660	

	CPU	0.000	5.302	fit succeeded

**3 IIIIIIIIIIIIJIIIIIIIIIIIIIIII**	R6X9	100.000^14	70.367^14	(66.686^14, 74.228^14)

	SSE	841284.881	8624.411	

	AIC	211.588	142.883	

	CPU	0.000	2.903	fit succeeded

#### Unoccupied a-sites

The space of 2088 models contains 99 models that do not have any terms with ratios of ATP bound per R1 > 1.5 (i.e. models consistent with ≤ 50% *a*-site filling in oligomers). Of the top 30 models, however, there were 14 such models, which is significant, 14/30 > 99/2088 with p < 3 × 10^-16^. In the reduced spaces of 1420 and 1287 models (of the previous paragraph), the proportions are 15/30 > 75/1420 and 13/30 > 54/1287, which also yield p < 3 × 10^-16^. Thus, this dataset does not support the existence of hexamers with >3 *a*-sites occupied by ATP. It should be noted that this statement implies a lack of *h*-site evidence if *a*-sites fill before *h*-sites.

**Figure 11:**
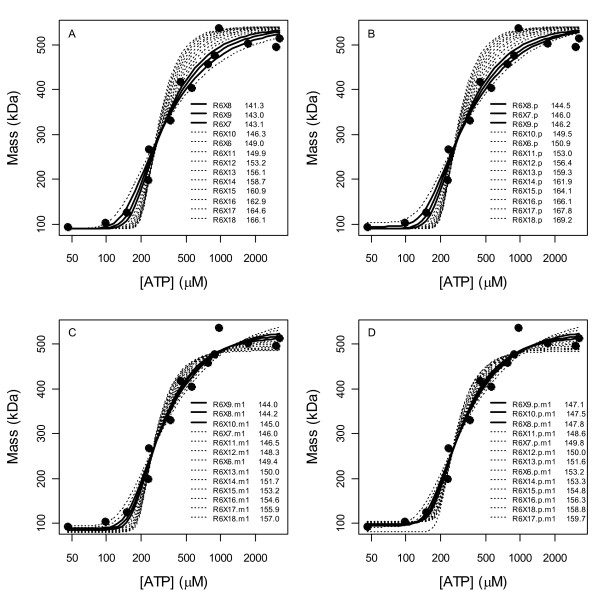
**The 13 single-edge spur models of Fig. 7(A) with *p *(B), M_1 _(C) or both (D) estimated**. The plots show that R^6^X^9 ^should perhaps be trusted more than R^6^X^8^. AICs in the B-D legends suggest that R^6^X^10 ^(which allows a 4^th ^filled a-site) is more likely than R^6^X^7^.

In the spaces of 1420 and 1287 models, the top 2 models are R^6^X^8 ^and R^6^X^9^. If the 13 single edge spur models are fitted with M_1 _of Eq. (6) estimated, a probability *p *that R1 is capable of oligomerizing estimated (in Eq. 5 *p *would then multiply [R_T_]), or both, the top 3 models are R^6^X^9^, R^6^X^8 ^and R^6^X^10 ^(see Fig. 11), i.e. the main claim is still supported but subsequent studies may find that a 4^th ^hexamer *a*-site can also be filled by ATP.

## Discussion

No terms higher than R^6^X^9 ^were needed to explain the ATP induced R1 hexamerization data found in figure 1 of reference [20]. If *s*-sites fill before *a*-sites, this implies that ~1/2 of the hexamer *a*-sites are not bound by ATP under the experimental conditions of this dataset. If *h*-sites fill after *a*-sites, this also implies that *h*-sites need not exist to explain this dataset. Since the *s*-site is at the dimer interface in yeast [26], and since it is reasonable that hexamers form as trimers of dimers, it is likely that *s*-sites do fill first.

If it is true that *s*-sites are always bound in oligomers and never bound in monomers, dNTP access to hexamer *s*-sites, as is needed for RNR control, implies that either the monomer-dimer-tetramer-hexamer equilibrium is rapid enough that changes in ligand bound to the *s*-site can occur via the monomer-dimer equilibrium, or perhaps the hexamer stabilizes internal dimers enough that hexamer *s*-sites can vacate without hexamer decomposition. The latter case would complicate the analysis as the term R^6^X^9 ^for example might then describe more than 3 filled *a*-sites.

Regarding *a*- before *h*-site filling, since *a*-sites are known to exist [16] and *h*-sites are in question, this is a reasonable default. The alternative, to assume *h*-site existence and instead challenge *a*-site existence, is much less reasonable.

**Figure 12:**
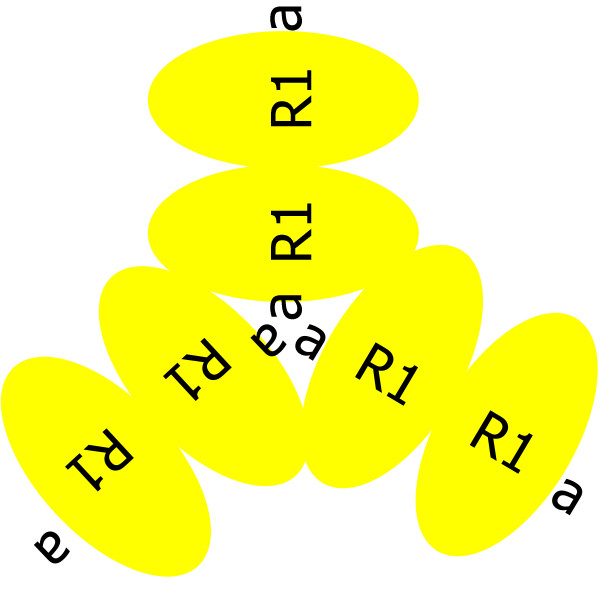
**R1 hexamer model**. R1 hexamer formation could result in the creation of two types of *a*-sites.

The most important short time constant (i.e. allosteric) feedback control of RNR is via dATP. This statement is based on ATP being too broadly used in cells for its level to be manipulated to control dNTP supply, and dTTP and dGTP being only selectivity controllers while when dATP controls selectivity, it also closes a large positive feedback loop that threads through dTTP and dGTP in series, see Fig 1). This may help dNTP pools fill uniformly and rapidly at the onset of S-phase. Once the dNTP pools are filled, dATP also has the responsibility of shutting off its *s*-site mediated positive feedback loop through *a*-site mediated inhibition of all four reaction rates (note that this argues in favor of dATP binding the *s*-site more tightly than the *a*-site). This picture suggests that prompt *a*-site mediated inhibition in response to changes in [dATP] is important since without a rapid response, the *s*-site mediated dATP positive feedback loop may cause [dATP] overshoot. Given that [dATP] << [ATP] implies that dATP collisions with *a*-sites are much rarer than ATP *a*-site collisions, when they do occur, it would help the circuit respond rapidly if at least half of the time the site was empty and thus ready to be filled. This leads to interesting speculations: R1 hexamers may have two types of *a*-sites, one for ATP and one for dATP, and beyond ligand differences, with this view (Fig. 12) dATP inhibition versus ATP activation could in part be due to differences in binding pockets of the two types of *a*-sites. Indeed, this may be the reason that R1 hexamers exist.

The approach used selects model terms (and thus parameters) based on how needed they are to explain the data analyzed. If, in solution, hexamers rarely have more than 3 ATPs bound to their *a*-sites, no parameters are allocated to complexes with higher numbers of bound ATP. The analysis presented does not claim that ≥ 3 *a*-sites will remain unoccupied under R1 crystallization conditions that may differ greatly from those used to generate the data analyzed here.

For the data analyzed, [R_T_] is 7 μM (i.e. yielding up to 21 μM of ATP binding sites if *h*-sites exist) and the minimum [X_T_] is 46 μM, so the approximation [X] = [X_T_] would not have been valid for this lowest [ATP] data point. The value of such approximations is less with ≥ 2 oligomerization states than with one (which has an analytic solution, see accompanying paper), as the univariate polynomial that results still requires a numerical solution (e.g. [X] = [X_T_] in Eq. (5) yields a 6^th ^order polynomial in free [R]), but univariate polynomials are solved much faster than multivariate polynomials, e.g. using ODEs as in Eq. (3), so the computational savings are worthwhile if the approximation is tolerable. For the data used here, as [X] = [X_T_] is valid for most of the data points, this approximation caused deviations of only 1% in the parameter estimates of Table 1 but it gave a 30-fold increase in computation speed.

Since there were 13, 286 and 3094 *K *infinity spur graphs with 1, 2 or 3 parameters, compared to 0, 2 and 15 *K *equality grid graphs, and since models with few parameters have an AIC advantage when dataset sizes are modest, it is not surprising that with 15 data points, the top models were all spur models. In the future, as automation affords richer datasets, grid graphs may become more competitive. Thus, though the grid graph enumeration efforts expended in this paper did not pay immediate dividends, they may in the future.

Contiguous stretches of equal binary *K *parameters within threads were not explored because binary *K *models were already non-competitive due to over-parameterization, and because additional ATP ligands on a *j*-mer would not have changed DLS masses detectably, so *K *cooperativity within threads would not have been detectable.

Microfluidic chip technology [28-31] will eventually enable 5-dimensional RNR studies where [ATP] and [NDP]s are fixed to *in vivo *levels and [R1], [R2], [dATP], [dTTP] and [dGTP] vary across ranges centered about physiological operating points. If ccems can automatically analyze new RNR data as it arrives, it could find uses in sequential experimental designs [32] where the chip conditions of the next measurements are determined in real time to implement efficient 5-D sampling strategies.

As protein expression and purification core facilities become more common, reconstituted network analyses where alleged protein-protein interactions are mathematically characterized for applications in systems biology [33,34] will eventually also become more common. It is anticipated here that many of these interactions will be combinatorially complex and that ccems will then find broader uses.

## Conclusion

No terms higher than R^6^X^9 ^were needed to explain the ATP induced R1 hexamerization data found in figure 1 of reference [20]. This suggests that under the experimental conditions of this dataset, ~1/2 of the hexamer *a*-sites are not bound by ATP, and that if *a*-sites fill before *h*-sites, that *h*-sites need not exist to explain this dataset.

The R package ccems currently solves 2-reactant problems where total reactant concentrations are known and manipulated, free reactant concentrations are determined by a system of mass action-based total concentration constraint polynomials, expected measurements are determined by model predicted complex concentrations, and the number of models is large due to combinatorial complexity. This is a generic *in vitro *synthetic biochemical system problem statement, so ccems could have a broad impact.

## Methods

Data were digitized by plotDigitizer [35] and analyzed using ccems[22]. Hessians of SSEs obtained using optim were divided by 2, inverted, multiplied by SSE/(*N *- *P*), and the square roots of the main diagonal were then multiplied by 1.96 to form 95% Wald CI. Parameters were estimated in exponentiated forms to constrain them to positive values.

## Competing interests

The author declares that he has no competing interests.

## Authors' contributions

TR performed the work and wrote the paper.

## Reviewer's comments

### Reviewer's report 1

Ossama B. Kashlan, University of Pittsburgh (nominated by Philip Hahnfeldt, Tufts)

As you've shown, you don't need to invoke the *h*-site to fit our figure 1 data. But we did need it for the other data in the paper, e.g. the global fit of DLS and activity data in figure 5 of our paper (with dTTP saturating the *s*-site). Since we wanted to use a single model for all the data, we therefore used an *h*-site to fit our figure 1 data. You should include a discussion of the potential of the *h*-site-less models to account for our figure 5 DLS data (as you noted, modeling activity data greatly increases the model space).

### Radivoyevitch's Response

**Figure 13:**
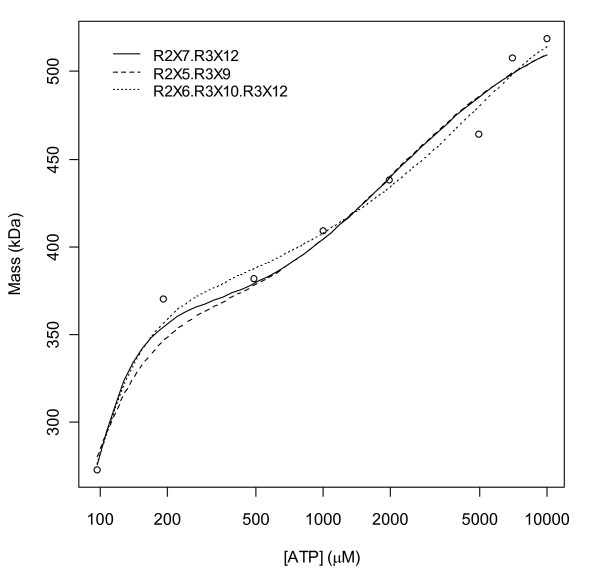
**Fits of the top 3 models with [dTTP] and [GDP] at saturating levels**. In response to reviewer Kashlan's comment, the top 3 models of the data in figure 5 of his paper are shown. All models yield m = 180 at [ATP] = 0; m = 181 was measured.

Let us denote by R the dimer complex (R1)_2_(GDP)_2_(dTTP)_2_. The full model is then(R1)

and in this case, the top 3 models are R^2^X^7 ^+ R^3^X^12^, R^2^X^5 ^+ R^3^X^9 ^and R^2^X^6 ^+ R^3^X^10 ^+ R^3^X^12^, i.e. models with *h*-site terms. Thus, you are indeed correct that there appears to be evidence for an *h*-site in your figure 5 (of ref [20]) data. Fits of these models are shown in the new Fig. 13. If I remove the three datapoints with [ATP] = 5 mM, 7 mM and 10 mM, i.e. values higher than those in your figure 1 data, similar results are obtained. Thus, perhaps the *h*-site is created by the presence of bound GDP substrate, or bound dTTP, or both, but yes, different inferences are drawn from your figure 1 and 5 datasets when they are analyzed individually. It should perhaps be noted that all of the tetramer terms above also have occupied *h*-sites, and that the model that you used did not, i.e. the methods presented may have value in analyses of your figure 5 data as well. Further, since no 1-parameter models were among the top 100, they likely all yielded poor fits.

### Kashlan's Response

As you concluded from the DLS data with saturating dTTP, an *h*-site is necessary to fit this data. However, I find your interpretation that the presence of GDP and/or dTTP possibly creates the *h*-site to be unlikely. More likely is that ligands bound to one site have heterotropic binding effects on the other sites. Under this logic, the underlying binding K's for ATP in the absence of GDP and dTTP are such that the DLS data under these conditions lack the complexity to require the invocation of an *h*-site. However, in the presence of GDP and dTTP, the underlying K's are such that this complexity is unmasked. I also have a few additional comments:

1. Regarding the assumption that *s*- fills before *a*- fills before *h*-site, and that oligomers always have certain sites filled. This assumption ignores a few important observations and possibilities. First is the ability of R1 to dimerize in the absence of a filled *s*-site, e.g. we observed that CDP reduction occurs (at a low rate) in the absence of an *s*-site ligand. Second, as you pointed out, is the ability to switch *s*-site ligands while the *a*- (and/or *h*-) site(s) are occupied. This is important, because you base your conclusion of a lack of *h*-site evidence on the fact that not enough ATP are bound to fill the *a*-sites. But unless *h*-site binding is dramatically weaker than *a*-site binding, binding the first few *h*-sites may be more favorable than filling the last few *a*-sites.

2. The minimal models presented as 'best', and from which physical conclusions are drawn, should be able to account for both (all) datasets. Having a different model framework for each given set of ligands adds a new level of complexity. Can you combine the AIC scores for the fits to both sets of data to find the best model(s), based at least on these data?

3. The conclusion on p. 3 should be edited to reflect the above comments and your response to my previous comment.

4. The background on p. 3 should be edited to read, "ATP binds to both of these sites and there is some evidence, based on RNR *mass and* activity versus [ATP] data,."

### Radivoyevitch's Response

Two comments: 1) if we let site creation include increasing the affinity so that an infinite K is now finite, we may be saying the same things; and 2) you have 9 data points in your figure 5 and 16 in your figure 1, and it would have been better to have these sample numbers reversed if indeed the conditions of your figure 5 yield more complex data. My inclination is to trust a 1-parameter model fitted to 15 points more than a 2-parameter model fitted to 9. Regarding your other two remarks:

1. By stating model assumptions I did not mean to say that I thought they must be true (no model is ever correct). What I meant to say is that my inferences are all conditional on their truth. The situation is such that unless such an assumption is made about the binding order, the meaning of a polynomial term that exists in a model is ambiguous. This is a big weakness, but I do not see any way around it. I now state this weakness at the end of the Limitations Section.

2. Note that we really have two different hub proteins for these two datasets, one with R as R1 monomer and the other with R as dTTP and GDP saturated R1 dimer. All complete K parameters downstream of these two hubs would thus be independent and there would thus be no way to pool the parameter estimates (beyond the error estimate). The joint model would thus be the sum of the terms.

3. The conclusion in the abstract has been softened to reflect conditionality on the experimental conditions of your figure 1 dataset.

4. Agreed, but this sentence no longer exists.

### Reviewer's report 2

Bin Hu, Los Alamos National Laboratory (nominated by William Hlavacek, LANL)

In this work, the author did a theoretical study on the equilibrium of ATP-induced hexamerization of the R1 subunit of ribonucleotide reductase (RNR), where combinatorial complexity is observed. Statistical hypotheses with assumptions were used to generate an array of models of the hexamerization process. Statistical comparison of the model structures suggests that a-sites may not be occupied by ATP in R1 hexamers at physiological ATP concentrations and the *h*-site may not exist. Results from this work suggest that the work in reference [20] did not consider the possibility of a-sites not being occupied, which allows the authors in [20] to suggest that the *h*-site exists. A final judgment about whether the *h*-site exists; however, can only be reached through additional experimental work. Although the statistical analysis approach used in this work is interesting, the author may wish to add a discussion comparing results from this work to those of Ref [20]. Minor comments are as follows:

1. An introduction to RNR, its function and regulation is needed. Currently there is only one sentence in the beginning of the Introduction about RNR.

2. It is not mentioned in the paper how the R1 subunit forms RNR with R2 units and what kind of multimers the R1 subunit can form and their biological importance. References, especially for the crystallography data, are needed.

3. In the Results section, the meaning of "complete dissociation constants" is not clear.

4. The R package ccems was first introduced without reference.

5. The author submitted two papers to this journal simultaneously. Instead of using "the accompanying paper," the author or editorial office may want to change it to some other description that may help readers to find out which paper the author is referencing.

6. I cannot tell whether the assumption "s-sites are always filled in oligomers and never filled in monomers" is acceptable in this study.

7. It would be interesting if the author compared the parameters used this work with those used in [20].

### Radivoyevitch's Responses

The model of ATP induced R1 hexamerization previously proposed by Kashlan *et al*. [20] assumed: a) that the binary dissociation constants *K *of the ATP binding sites *s*, *a *and *h *are the same within oligomers (within site types); b) that these *K*_s_, *K*_a _and *K*_h _are infinite in structures smaller than dimers, tetramers and hexamers, respectively (note that all three are thus infinite in monomers); c) that finite *K *are equal wherever it is plausible that they might be, i.e. that *K*_a _in tetramers equals *K*_a _in hexamers and that *K*_s _is the same across dimers, tetramers and hexamers; d) that the dissociation constants for R1 binding to itself (*K*_R_R_), R1 dimers binding to themselves (*K*_R2_R2_), and R1 tetramers binding to R1 dimers (*K*_R4_R2_), are independently adjustable; and e) that R1 tetramers can isomerize with an isomerization constant *K*_is_. Assumptions a) to c) constrain the model and d) to e) broaden it. When Kashlan *et al*. fitted their model to their DLS data, *K*_R_R_, *K*_R2_R2_, *K*_R4_R2 _and *K*_is _were treated as being independent of R1 ligands, and consistent with these assumptions, the data in their figures 1 and 3-9 were fitted to single values of these constants such that the fits in these figures did not appear too poor). With respect to their figure 1 data, however, the first five residuals of their fit were negative and thus correlated, and although the residuals were small and thus subtle, the fit was thus poor. This paper focuses on their figure 1 data alone.

Regarding proof that an h-site does or does not exist, I agree that binding studies must be performed to see how many ATPs actually bind to R1, but such studies may be difficult, as evidenced by the fact that they have not yet been performed. My hunch is that experimental challenges are associated with weak ATP binding and thus the high [ATP] needed to achieve binding, which may make changes in free [ATP] due to free ATP losses to ATP bound to R1 difficult to detect. Regarding comparisons of results, since the single model that they fitted to their DLS data was also based on their RNR activity data, and since activity data is much more complicated to analyze than mass data because conjectures about different activity parameters being zero or equal to each other greatly expands the model space further, and since my software is not yet ready for more than one type of oligomer in activity data analyses (in the accompanying paper the enzyme TK1 was strictly tetrameric), our results cannot be compared. Regarding your points:

1. Background material regarding dNTP supply and RNR have now been added to the Introduction. However, since I do not model RNR activity data, this work has limited relevance to dNTP supply metabolism. My focus is thus on the methods developed. Indeed, it may be best to view R1 as merely some protein that has either 2 or 3 binding sites on it for a ligand that induces its hexamerization.

2. A) R2 is irrelevant here since this paper does not delve into activity data and since R2 was not present in the experiment that yielded the DLS average mass data analyzed. B) The first eukaryotic (yeast) R1 structure showed a dimer and this was referenced [26]. Though a dATP induced R1 tetramer was observed in Ref [20], it was not observed in [27], and no lab has observed it directly using the more relevant ligand (for this paper) ATP. Thus, tetramers could perhaps have been left out of the model space, but there is strong support for R1 monomers (e.g. the low [ATP] DLS data in Fig. 7), dimers (the structure in [26]) and hexamers (e.g. the high [ATP] DLS data in Fig. 7).

3. By complete dissociation constants I mean those where the Gibbs Free energies are with respect to all reactants being completely separated from one another by infinite distances. In contrast, by binary dissociation constants I mean situations where only one reactant (or perhaps a subcomplex) is separated out at infinity while all of the other reactants remain bound together.

4. The link to my ccems page is now referenced earlier.

5. They should end up back-to-back and I hope readers will read, and know of, both.

6. The conclusions remain the same if I drop *h*-site terms and blow up the model space by introducing s-site terms, i.e. there is some support for the assumption besides dTTP induced R1dimerization results in [25] and the structure in [26].

7. The model used in [20] has 7 parameters. Their binary K values for ATP binding were 100 μM for dimers and tetramers and 1.1 mM for hexamers. For R1 oligomerization and isomerization their values were *K*_R_R _= 170 μM, *K*_R2_R2 _= 2-5 mM, *K*_R4_R2 _= 2-6 mM and *K*_is _= 10-40 where ranges depend on different tetrameric activity assumptions. Without the same parameters in my models, comparisons are difficult. The best model in Table 1 is R6X8 and it has a geometric mean binary binding constant of 63 μM. Since all of the binary K values of [20] are ≥ 100 μM, their geometric mean must also be ≥ 100 μM. Indeed, aassuming ATP fills 6 s-sites and 2 a-sites in R6X8, one obtains a geometric mean binary K of 190 μM = ((100)^8^(170)^3^(3000)^1^(3000)^1^)^1/13^, i.e. there is a difference of a factor of 3 in parameter estimates between analyses.

### Reviewer's report 3

#### Rainer K. Sachs, UC Berkeley

In general, is there some systematic rationale and/or underlying reasoning on what criteria to use to distinguish hypotheses? Do you estimate that almost any criterion would give the same final answers qualitatively? How did you decide on 30 models in your comparison of model proportions? What is your main motivation to study RNR? Can your software handle more than two reactants? Can you provide a code use example?

### Radivoyevitch's Responses

The AIC was used (without consideration of alternatives such as the Bayesian Information Criterion, BIC) only because it is the most popular. The idea was to pick a criterion to present my main contribution, which is in model space generation rather than model selection. Though there may be reasons to switch to a different criterion that I have yet to learn of, in the interim, the AIC is my default. I suspect that the conclusions made here would be robust to such changes.

The choice of 30 models involved data snooping as you may have guessed, i.e. 30 looked like a good breaking point for a claim that the data does not support an *h*-site. Thus, the difference in proportions p-value that I reported may be overly significant. Nevertheless, the Kolmogorov-Smironov Test is with respect to entire distributions in Fig. 6, so the conclusion that this particular dataset does not demand the existence of an *h*-site is robust. There is no indication that this conclusion will hold under different experimental conditions, however, e.g. see Kashlan's review above.

**Figure 14:**
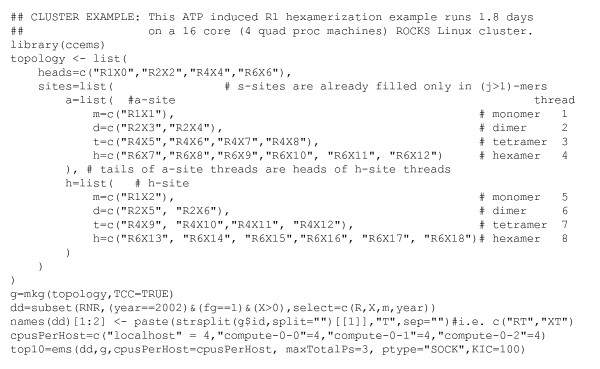
**ccems code example**. These codes generate Table 1 and the RX model space used in this paper.

Three paragraphs were added to the Introduction to motivate RNR research. The automated model space generation capabilities of ccems are indeed limited to two reactants and this is now stated in the title and elsewhere. If R and ccems are installed, the R command load(ccems) followed by ?ccems yields help which includes the code example in Fig. 14 which can be pasted into the R command line to create the model space used here.
